# Sodium Coupled Bicarbonate Influx Regulates Intracellular and Apical pH in Cultured Rat Caput Epididymal Epithelium

**DOI:** 10.1371/journal.pone.0022283

**Published:** 2011-08-22

**Authors:** Wu-Lin Zuo, Sheng Li, Jie-Hong Huang, Deng-Liang Yang, Geng Zhang, Si-Liang Chen, Ye-Chun Ruan, Ke-Nan Ye, Christopher H. K. Cheng, Wen-Liang Zhou

**Affiliations:** 1 School of Life Sciences, Sun Yat-sen University, Guangzhou, China; 2 School of Biomedical Sciences, The Chinese University of Hong Kong, Shatin, N.T., Hong Kong, China; Abramson Research Center, United States of America

## Abstract

**Background:**

The epithelium lining the epididymis provides an optimal acidic fluid microenvironment in the epididymal tract that enable spermatozoa to complete the maturation process. The present study aims to investigate the functional role of Na^+^/HCO_3_^−^ cotransporter in the pH regulation in rat epididymis.

**Method/Principal Findings:**

Immunofluorescence staining of pan cytokeratin in the primary culture of rat caput epididymal epithelium showed that the system was a suitable model for investigating the function of epididymal epithelium. Intracellular and apical pH were measured using the fluorescent pH sensitive probe carboxy-seminaphthorhodafluor-4F acetoxymethyl ester (SNARF-4F) and sparklet pH electrode respectively to explore the functional role of rat epididymal epithelium. In the HEPES buffered Krebs-Henseleit(KH) solution, the intracellular pH (pHi) recovery from NH_4_Cl induced acidification in the cultured caput epididymal epithelium was completely inhibited by amiloride, the inhibitor of Na^+^/H^+^ exchanger (NHE). Immediately changing of the KH solution from HEPES buffered to HCO_3_^−^ buffered would cause another pHi recovery. The pHi recovery in HCO_3_^−^ buffered KH solution was inhibited by 4, 4diisothiocyanatostilbene-2, 2-disulfonic acid (DIDS), the inhibitor of HCO_3_^−^ transporter or by removal of extracellular Na^+^. The extracellular pH measurement showed that the apical pH would increase when adding DIDS to the apical side of epididymal epithelial monolayer, however adding DIDS to the basolateral side had no effect on apical pH.

**Conclusions:**

The present study shows that sodium coupled bicarbonate influx regulates intracellular and apical pH in cultured caput epididymal epithelium.

## Introduction

After the generation in the testis, the sperm enter the tract of epididymis and complete the maturation process [1]. The epididymal epithelium creates a mature and stable microenvironment, including the reabsorption of majority of the testis fluid, Na^+^ reabsorption [2], the secretion of K^+^ [3] and Cl^−^ [2], [4] and the acidification of the microenvironment in the epididymal tract [5], so that the sperm obtain the capability of approaching eggs and fertility [6].

The low concentration of HCO_3_^−^ and low pH value are important to keep sperm storing in the epididymis in a quiescence state to complete maturation. In previous studies, Na^+^/H^+^ exchanger 1 (NHE1) was found on the basolateral side of all the epididymal epithelial cells along the entire epididymal tract and NHE2 was detected on the apical membrane of principle cells in all regions of epididymis (caput, corpus and cauda epididymis) except the initial segments [7]. It indicates that NHE2 may take part in the Na^+^ reabsorption and acidification in the epididymal tract. However, there are discrepancies on the expression of NHE3 in epididymis [8], [9]. Except that NHE participates in the tract acidification, another candidate which may participate in the epididymal tract acidification is the H^+^-ATPase. In the epididymis, H^+^-ATPase is highly expressed on the apical plasma membrane of narrow and clear cells in each segment of epididymis, as well as the vesicle membrane in the cells [10], [11]. Furthermore, from the proximal end to distant end of epididymis, the expression level generally increases as the number of H^+^-ATPase dramatically rises [12]. It is believed that the specific expression of H^+^-ATPase on the apical membrane of the clear cells is vital for the luminal acidification, especially for the caudal epididymis and the proximal end of vas deferens [12], [13].

Not only H^+^ secretion, but also HCO_3_^−^ reabsorption may regulate the luminal acidic microenvironment. Previous studies show that epididymis, especially its proximal segment, has a strong ability of water reabsorption, companied with the reabsorption of Na^+^ and HCO_3_^−^ [13], [14], [15]. Many types of carbonic anhydrase (CA) enzymes, which related to the function of Na^+^/HCO_3_^−^ cotransporters (NBC), are also highly expressed at caput epididymal epithelium [13], [16]. These indicates NBC, especially at the proximal region, may involve in the HCO_3_^−^ reabsorption. The NBC belongs to a gene family called solute carrier 4A (SLC4A). They are divided into two groups: electrogenic NBC and electroneutral NBC [17]. NBC distributes in almost all the tissues and cells whose main function is pH regulation and ion reabsorption [18]. Several subtypes of NBC have already been found on epididymal epithelium, NBCe1-A is expressed on the basolateral membrane of all the epididymal epithelium and highly expressed in the proximal segment [19]. NBC3 is expressed in the narrow cells of the caput epididymis, with less expression in the clear cells of corpus and caudal epididymis [20]. Though previous literatures reported some subtypes of NBC existed on epididymal epithelium, the functional role of NBC in regulating the luminal proton/base transport in epididymis is still less known. Therefore, we focus on caput epididymal epithelium and found that the NBC takes part in the regulation of intracellular pH(pHi) and the reabsorption of apical HCO_3_^−^.

## Results

### Characterization of primary culture of rat caput epididymal epithelium

**Figure 1:**
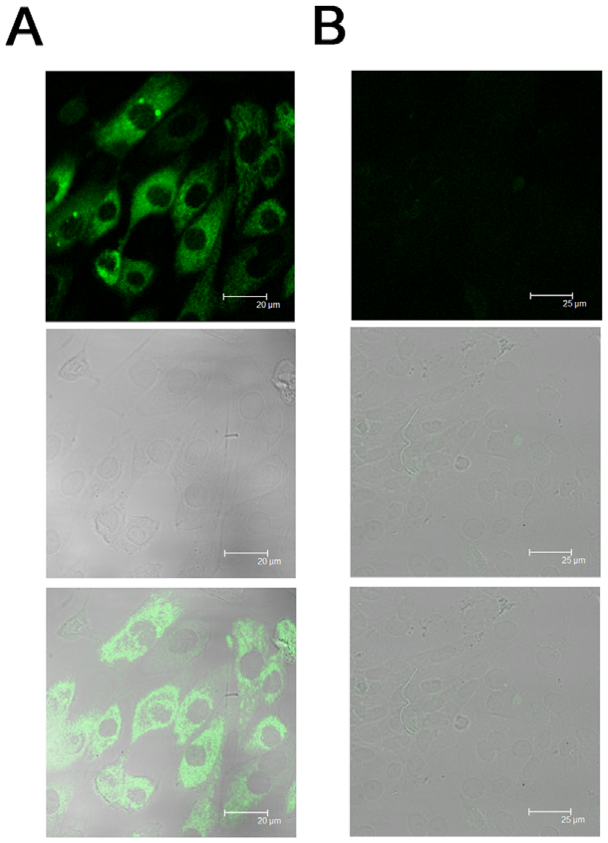
Establishment of primary culture of rat caput epididymal epithelium. A: In the above is the fluorescence image of cultured rat caput epididymal epithelial cells showing FITC immunoreactivity for pan keratin, in the middle is bright field image of caput epididymal epithelial cells and in the below is overlapping image of the fluorescence and bright field images; B: In the above of native control group is the fluorescence image of cultured rat caput epididymal epithelial cells without the application of primary antibody, in the middle is bright field image of caput epididymal epithelial cells and in the below is overlapping image of the fluorescence and bright field images.

To study the functional role of caput epididymal epithelium, the present study used the anti pan cytokeratin antibody in immunofluorescence and found that most of cultured cells expressed pan cytokeratin, the marker for epithelial cells (Fig. 1).

### HCO_3_^−^ participate in intracellular acidification recovery

**Figure 2:**
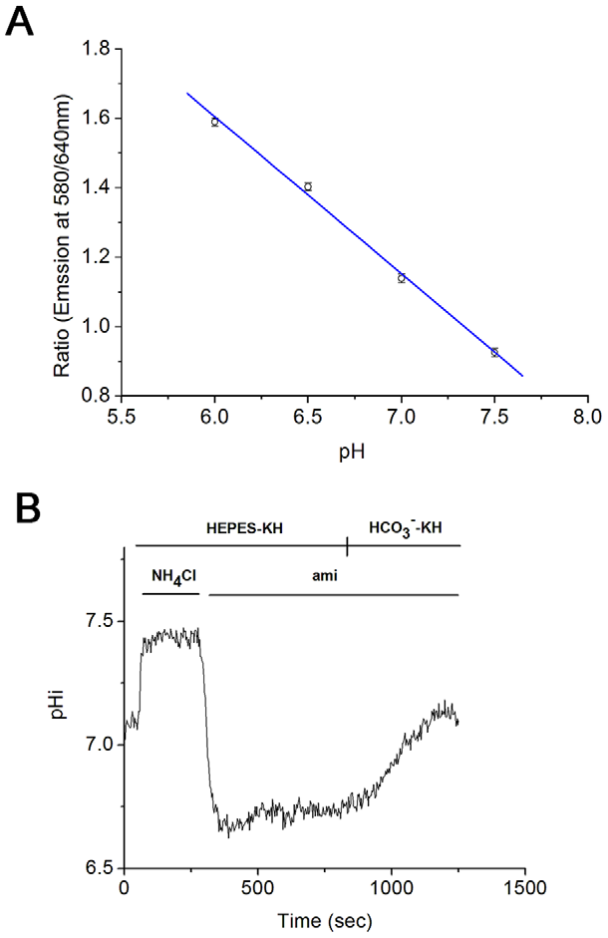
HCO_3_^−^-induced intracellular acidification recovery in rat caput epididymal epithelium. A: pHi calibration linear of rat caput epididymal epithelium; B: Representative record chart showing the function of HCO_3_^−^ in NH_4_Cl induced acidification recovery in rat caput epididymal epithelium. In the HEPES-KH solution, 40 mM NH_4_Cl induced intracellular alkalization. Then, in the HCO_3_^−^ free condition containing 1 mM amiloride induced transient intracellular acidification, and the recovery was inhibited. After that, the cells were perfused with HCO_3_^−^-KH solution with 1 mM amiloride, and the intracellular acidification was recovered. NH_4_Cl, 40 mM NH_4_Cl; ami, 1 mM amiloride; Each point represents the mean ±S.E.M. n = 4.

To study the intracellular pH (pHi) regulation in caput epididymal epithelium, we performed pHi measurements. First, high K^+^/nigericin method was applied to calibrate the pHi of caput epididymal epithelium. The pH values of calibration were set as 6.0, 6.5, 7.0 and 7.5 respectively. We recorded the ratios of intracellular fluorescence intensity of different emission wavelengths which represent the pHi values and made the linear calibration (Fig. 2A).

In basal level, the pHi of rat caput epididymal epithelium is 7.122±0.045 (n = 8). Rat caput epididymal epithelium was immersed in HEPES buffered KH(HEPES-KH) solution. Transient application of 40 mM NH_4_Cl would induce the swift increase of epithelial pHi. However, washing out 40 mM NH_4_Cl with HEPES-KH solution would induce epithelial transient acidification, and then a pHi recovery was observed from relatively acid level to basal level (data not shown). According to previous studies, the recovery is mainly mediated by NHE, which transports Na^+^ into the cell and exports H^+^ to keep the stabilization of pHi [9], [21]. Application of 1 mM amiloride, the specific inhibitor of NHE, would inhibit most of the recovery and it supports that the recovery is mainly mediated by NHE (Fig. 2B). However, if HCO_3_^−^ was supplied by changing the solution into the HCO_3_^−^ buffered KH(HCO_3_^−^-KH) solution containing 1 mM amiloride, a pHi recovery from acid level to basal level could be recorded (Fig. 2B). The recovery rate that represents the working efficiency was 0.078±0.012 pH unit/min (n = 4). The results stated above showed that, besides NHE, HCO_3_^−^ could play a critical role for pHi recovery from acid level to basal level.

### Intracellular acidification recovery mediated by HCO_3_^−^ is Na^+^ dependent

**Figure 3:**
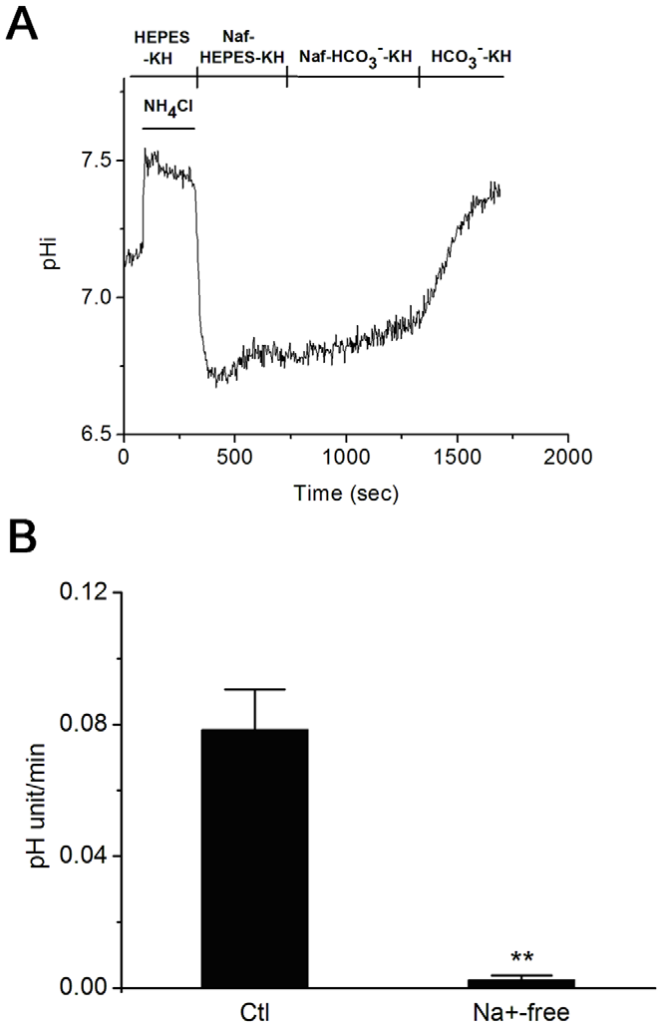
Na^+^ dependent acidification recovery in rat caput epididymal epithelium. A: Representative record chart showing involvement of Na^+^ in the HCO_3_^−^ mediated recovery for NH_4_Cl induced acidification in rat caput epididymal epithelium. The HEPES-KH solution containing 40 mM NH_4_Cl caused intracellular alkalization. Washing the NH_4_Cl with Naf-HEPES-KH solution would arouse intracellular acidification with no remarkable recovery. When the perfusion solution was changed into Naf-HCO_3_^−^-KH solution, the recovery was still depressed. When the perfusion solution was changed into normal KH solution, the intracellular acidification recovered to basal level; B: The summary of the results showing the effect of Na^+^ on HCO_3_^−^ mediated intracellular acidification recovery. HCO_3_^−^ mediated intracellular acidification recovery in Fig. 2B is set as control group. Data are expressed as the change of the pH value per minute. NH_4_Cl, 40 mM NH_4_Cl; ami, 1 mM amiloride; Naf, Na+ free; Ctl, control; Columns and error bars are mean ± S.E.M. (n = 3; ** P<0.01 vs Ctl).

Rat caput epididymal epithelium was treated with Na^+^-free & HCO_3_^−^ buffered KH(Naf-HCO_3_^−^-KH) solution and Na^+^-free & HEPES buffered KH(Naf-HEPES-KH) solution to test that whether Na^+^ takes part in the NH_4_Cl induced intracellular acidification recovery. The cells were first immersed in the HEPES-KH solution. Then, 40 mM NH_4_Cl was applied and then washed by Naf-HEPES-KH solution to induce the intracellular transient acidification (Fig. 3A). However, the acidification went without significant recovery, suggesting the exclusion of Na^+^ inhibited the activity of NHE. When changing with Naf-HCO_3_^−^-KH solution, the acidified pHi did not significantly increase. The intracellular acidification recovery was significantly inhibited in the Na^+^-free solution, even when HCO_3_^−^ existed in the solution. The recovery rate decreased to 0.003±0.001 (n = 3; Fig. 3B) which significantly differed from that control recovery rate induced by HCO_3_^−^ (Fig. 2B). The intracellular pH value of the cells presented a remarkable recovery (Fig. 3A) when perfused with HCO_3_^−^-KH solution containing Na^+^, showing the viability and integrity of the cells. The results mentioned above show that HCO_3_^−^ mediated intracellular acidification recovery is Na^+^-dependent.

### DIDS inhibits intracellular acidification recovery

**Figure 4:**
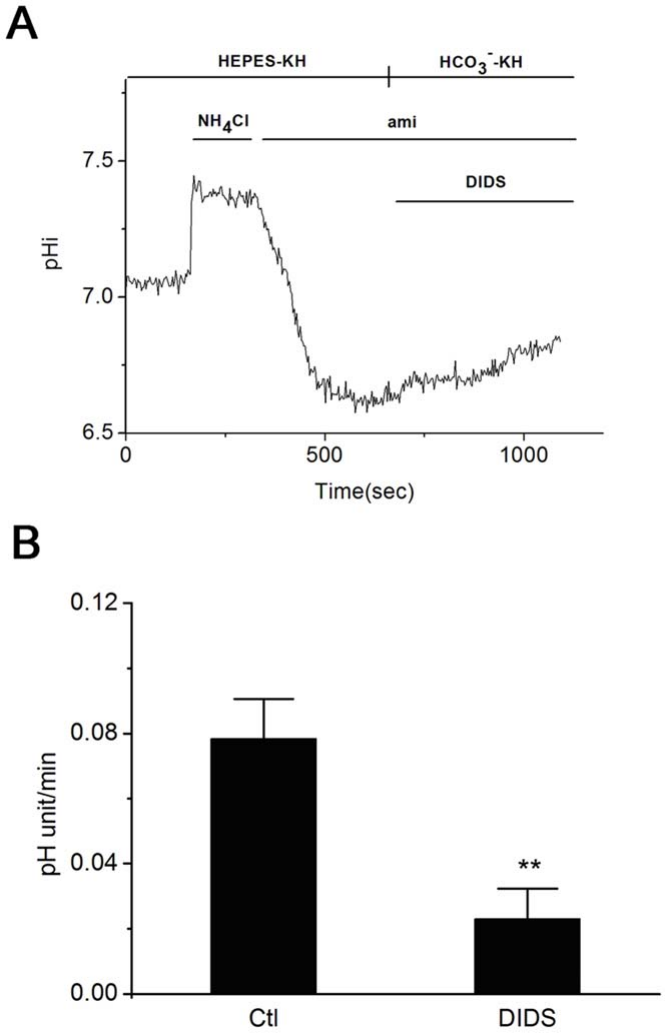
DIDS inhibits HCO_3_^−^ induced intracellular acidification recovery in rat caput epididymal epithelium. A: The record chart showing that DIDS inhibited the HCO_3_^−^ mediated recovery for NH_4_Cl induced acidification in the rat caput epididymal epithelium. The HEPES-KH solution containing 40 mM NH_4_Cl caused intracellular alkalization. Wash the NH_4_Cl with HEPES-KH solution with 1 mM amiloride would arouse intracellular acidification with no remarkable recovery. The recovery was still suppressed when the solution was changed into HCO_3_^−^ -KH solution containing 1 mM DIDS and 1 mM amiloride; B: The summary of results showing the effects of DIDS on the HCO_3_^−^ mediated intracellular acidification recovery. HCO_3_^−^ mediated intracellular acidification recovery in Fig. 2B is set as control group. Data are expressed as the change of the pH value per minute. NH_4_Cl, 40 mM NH_4_Cl; ami, 1 mM amiloride; DIDS, 1 mM DIDS; Ctl, Control; Columns and error bars are mean ± S.E.M. (n = 5; **P<0.01 vs Ctl).

To verify whether bicarbonate transports are involved in HCO_3_^−^ mediated intracellular acidification recovery, 1 mM DIDS, the specific inhibitor for bicarbonate transporters, was applied to HCO_3_^−^ buffered KH solution. Application 40 mM NH_4_Cl following the washing induced transient intracellular acidification. 1 mM amiloride was applied into the solution to exclude the effect of NHE. Perfusion of HCO_3_^−^-KH solution containing 1 mM DIDS and 1 mM amiloride could not induce remarkable recovery (Fig. 4A). Compared with the control group, 1 mM DIDS markedly depressed the recovery and the recovery rate was 0.023±0.009 (n = 5; Fig. 4B). These data show that bicarbonate transporter contributes to the intracellular acidification recovery. Since Na^+^ also participates in HCO_3_^−^ mediated intracellular acidification recovery, the intracellular acidification recovery may mediated by NBC.

### NBC at the apical membrane of rat caput epididymal epithelium regulate apical pH

**Figure 5:**
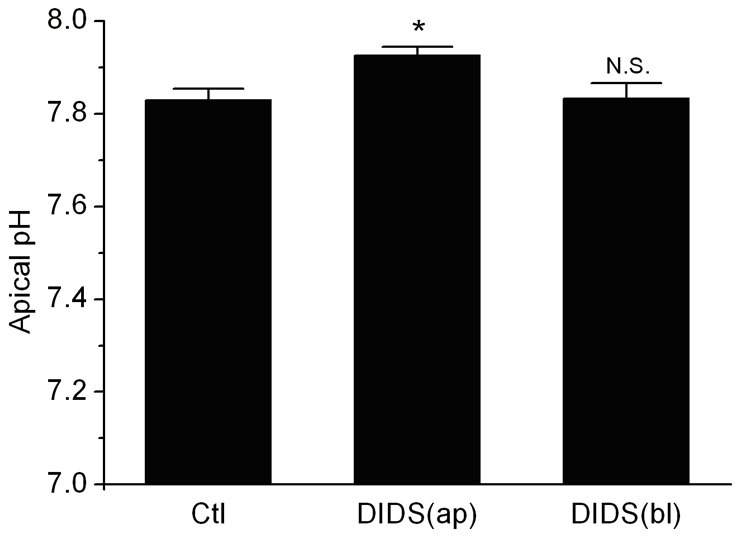
NBC on the apical membrane of rat epididymal epithelium regulates epididymal apical pH. In the control group, the cultured rat caput epididymal epithelium was immersed in Cl^−^ free KH solution without any treatment of DIDS (n = 7). 1 mM DIDS was applied to the apical(n = 3) or basolateral(n = 3) side of the cultured epithelial monolayer and incubated for 1 hour. Ctl, Control; DIDS, 1 mM DIDS; ap, apical; bl, basolateral; N.S., no significant difference vs Ctl; All bars represent mean ± S.E.M. (*P<0.05 vs Ctl).

To investigate whether the NBC regulates the fluid microenvironment of epididymal tract, the extracellular pH was measured. After 4 days culture in transwell, the caput epididymal epithelial monolayer formed and the medium of apical and basolateral sides of the caput epididymal epithelium were changed into Cl^−^ free KH solution (pH = 7.400). The transwells without cells were set as the negative control. For the negative control, there is no significance difference after 1 hour incubation (pH = 7.392±0.010, n = 4) or adding DIDS (initial pH = 7.410±0.010, n = 4; after 1 h incubation, pH = 7.393±0.013, n = 4). After incubation at 32°C for 1 hour, the pH value of the solution at the apical side was 7.830±0.024(n = 7), while incubating with 1 mM DIDS at the apical side would increase the pH to 7.927±0.018 (n = 3; Fig. 5). The results show that the pH of the apical side significantly increased compared with that in the control group without 1 mM DIDS, indicating that NBC might participate in the regulation of epididymal apical pH. When applied 1 mM DIDS at the basolateral side, the pH value of the solution at basolateral side was 7.833±0.033(n = 3; Fig. 5), which did no significantly change compared with that in the control group. The results above imply that NBC located at the apical side but not basolateral side of the caput epididymal epithelium could regulate epididymal apical pH.

## Discussion

Epididymal epithelium provides a desirable microenvironment by the regulation of ion transport for sperm storage, so that sperm could quiescently achieve maturation. Through the *in vivo* or *in vitro* perfusion of physiological solution in rat epididymis, Wong PY found that the reabsorption of Na^+^ drove the reabsorption of water [22], [23] by NHE located at the apical membrane of epididymal epithelium [7], [21]. A generally increase of K^+^ concentration was also found in epididymal tract that may be mediated both by an ATP-activated cation channel [3] and a Ca^2+^-activated K^+^ channel [24]. In addition, the extracellur pH decreases through the epididymal tract and the acidification of the tract is caused by H^+^ pumped into the epididymis tract via H^+^-ATPase distributed at the apical membrane of epididymal clear cells [10]. Moreover, the concentration of HCO_3_^−^ in epididymal tract is far lower than that in blood [25]. Morphological evidences show that the epididymis epithelium express various bicarbonate transporters, such as NBC [19] and anion exchanger(AE) [26], indicating that the epididymal epithelium obtains a powerful capability of HCO_3_^−^ reabsorption, but the function of HCO_3_^−^ transportation is still unknown. In this study, to figure out the functional role of HCO_3_^−^ transportation in epididymal epithelial cells, we first used a revised culture approach to achieve more and purer epididymal epithelium. Considering swimming sperm would disturb the adherence of epididymal epithelium, the present study chose 5 weeks SD rat without sperm in epididymis as the animal model. As SD rats have matured sexually 6 weeks after birth, we adopted SD rats in the age of 5 weeks, which are practically closed to the matured status. Immunocytochemistry experiments showed that the majority of the cultured cells expressed pan keratin, a marker for epithelial cells, indicating that this culture system is suitable for studying the function of epididymal epithelium.

pHi exhibits an extensive function during physiological process. Not only could it regulate the proliferation and differentiation of the cells [27], [28], but also it could function in immunological defense [29] as well as regulate ion channel activity. Mammals keep pHi in a stable value via different transporters on the cell membrane. These transporters include the NHE family (Exports intracellular H^+^), the Na^+^-dependent and Na^+^-independent bicarbonate transporters family (Transport HCO_3_^−^), and H^+^-ATPase (Transports H^+^ by consumption of ATP). Functional study shows that NHE1 participate in the metabolism regulation of pHi in epididymal epithelium [21], although there are discrepancies on the expression of NHE, functional studies showed that NHE3 distributing at the apical membrane of the proximal end of epididymis regulated the pHi so as to modulate the luminal pH of epididymis [9], [21]. However, controversies exist in the functional study of NHE2 that presents in the caput, corpus, and caudal regions but is absent in the initial segment [7], [13]. In addition to the NHE family, recent data from our group showed that H^+^-ATPase located on cultured epididymal clear cells could also regulate the pHi and luminal pH of the epididymal fluid via secretion of H^+^ towards epididymal luminal side [30], which consist with morphological study. In the present study, the cultured caput epididymal epithelium was found to have a HCO_3_^−^ dependent pHi acidification recovery in the presence of amiloride, the NHE inhibitor, suggesting that there is a HCO_3_^−^ dependent pathway regulating the pHi in the epididymal epithelium besides NHE. The NH_4_Cl induced acidification recovery was markedly suppressed when applying Na^+^-free KH solution with HCO_3_^−^, indicating that the HCO_3_^−^ dependent acidification recovery is Na^+^ dependent. NBC, a Na^+^ dependent bicarbonate transporter, may mediate the acidification recovery. With low concentration, DIDS serve as the inhibitor of some anion channels, such as Ca^2+^- sensitive [31] and volume-sensitive Cl^−^ channels [32]. While in high concentration, DIDS is always set as the inhibitor of HCO_3_^−^ transporter, including Na^+^/HCO_3_^−^ cotransporter [33], [34] and AE [35]. The involvement of NBC was next testified by treatment of 1 mM DIDS, the NBC inhibitor which significantly inhibited the HCO_3_^−^ and Na^+^ dependent acidification recovery.

Sodium dependent bicarbonate cotransporters play biological function in ion transport, regulation of intracellular and extracellular acid/base equilibrium, maintaining cell volume and other cellular processes [36]–[39]. Previous study showed that NBC3 only expressed on the apical/clear cells, the minor cell population, in different regions of epididymis [20]. In the present study, we found that almost all the cultured caput epididymal epithelium exhibit a Na^+^-dependent HCO_3_^−^ influx. Since over 80% of epididymal epithelium are classified as principal cells [40], we deem that, at least the principal cells contributed to the Na^+^ coupled HCO_3_^−^ influx in order to regulate intracellular and luminal pH. Removal of Cl^−^ in the solution had no effect on this HCO_3_^−^ influx (data not shown), thus exclude the participation of Na^+^ driven Cl^−^/HCO_3_^−^ exchanger. Therefore, Na^+^/HCO_3_^−^ cotransporter having a 1∶2 stoichiometry may be a candidate for mediating HCO_3_^−^ influx. Achievement of further morphological evidence requires more endeavors in the research.

To study the influence of intracellular pH on epithelial extracellular pH, we performed extracellular pH measurement. There are many ion transports expressed on the epididymal epithelium, including AE2. AE2 that takes Cl^−^ into the cell and export HCO_3_^−^ out of the cytoplasm, is expressed on the basolateral side of epididymal epithelium [26]. High concentration of DIDS also inhibit AE2. In order to exclude the effect of AE2 on the extracellular pH measurement, the Cl^−^ in the solution was removed. At the same time, the Cl^−^ free KH solution could also inhibit Na^+^/K^+^/2Cl^−^ cotransporter, which pumps Na^+^ into the cytoplasm. Without intracellular Na^+^, the NBCe1_A that is expressed on the basolateral side of epididymal epithelium [19] and export Na^+^ and HCO_3_^−^ out of the cytoplasm, is inhibited. All in all, the role of Cl^−^-free KH solution is to exclude the effects of other HCO_3_^−^ transporters on the extracellular pH measurement. Due to the inhibition of the HCO_3_^−^ transporters on the basolateral side, the pH of apical medium increased from 7.4 to about 7.8. Our results show that pretreatment of DIDS on apical but not the basolateral membrane of cultured epididymal monolayer rendered higher pH value of apical fluid, indicating that the inhibition of the function of apical NBC would prevent the reabsorption of HCO_3_^−^, leading to the alkalization of epididymal apical fluid environment. Although the experimental solution in experiment *in vitro* had larger volume than in vivo epididymal tract's fluid, the difference in volume was insufficient to counteract the significant discrepancy of pH value. In light of foregoing, the present in vitro experiment testified that NBC on the apical side participates in the regulation of apical pH.

In conclusion, the present study has revealed that Na^+^ coupled bicarbonate influx existed in caput epididymal epithelium, which could not only regulate pHi of epididymal epithelium, but also modulate epididymal luminal microenvironment. These features provide insight into the physiological role of epididymis and exert profound influence on sperm maturation.

## Materials and Methods

### Ethics Statement

All animal procedures in this study were approved by the Animal Experimentation Ethics Committee of School of Life Sciences, Sun Yat-sen University.

### Solutions

The content of phosphate buffered saline (PBS) in immunohistochemistry is as followed (concentration unit is mM) NaCl 154, KH_2_PO_4_ 1 and Na_2_HPO_4_^.^7H_2_O 2.96, pH = 7.4. The KH solution in intracellular and extracellular pH measurements had the following compositions (concentration unit is mM) : NaCl 117, KCl 4.7, CaCl_2_ 2.56, MgSO_4_^.^7H_2_O 1.2, NaHCO_3_ 24.8, KH_2_PO_4_ 1.2 and D-Glucose 11.1, pH = 7.4. In some experiments, HCO_3_^−^ was replaced by 10 mM HEPES, Na^+^ was replaced by equal NMDG and Cl^−^ was replaced by equal gluconate.

### Cell culture

Five weeks Sprague-Dawley rats were purchased from the Guangdong Medical Laboratory Animal Center. The procedures of caput epididymal epithelium cell culture were revised from the previous protocol [41], [42]. Considering that swimming sperm would disturb the adherence of epididymal epithelium, we chose immature rat with no sperm in epididymis in the present experiments. Male Sprague-Dawley rats, 5 weeks and weighing 100–120 g were sacrificed by 100% CO_2_. The part below the abdomen of the rat was split using surgical instruments, and the tissue of testis and epididymis were dissected out by forceps. After the adipose wrapped around epididymis was removed, the tissue of caput epididymis was immersed in Hanks Buffered Salt Solution (HBSS) (Gibco Laboratories, Grand Island, NY). Being washed by HBSS three times, the caput epididymal tissue was finely chopped by scissors. Following is to apply 0.125% (w/v) trypsin (Gibco Laboratories, Grand Island, NY) digested for 40 minutes by vigorous shaking. The tissue was separated by centrifugation at 800 g for 5 minutes. The supernatant was discarded and the cells were transferred into 0.05% (w/v) collagenase (Sigma Chemical Co., St. Louis, MO) for 30 minutes digestion with shaking. The cells were further isolated by centrifugation at 800 g for 5 minutes and the supernatant was discarded. The cells were suspended in Eagle Minimum Essential Medium (EMEM) culture medium (Gibco Laboratories, Grand Island, NY) containing nonessential amino acid (0.1 mM), sodium pyruvate (1 mM), 5α-DHT (1 nM), 10% FBS(v/v), penicillin (100 IU/ml), and streptomycin (100 µg/ml) and incubated in a flask at 32°C in a humidified atmosphere of 5% CO_2_/95% O_2_. After 5–6 hours of incubation, the non-epithelial cells, such as fibroblasts and muscle cells would attach to the flask inside surface, but the epithelial cells were unattached and free in the suspension. Thus the epithelial cells could be separated from the non-epithelial cells by transferring the cell suspension to glass coverslips (Superior Marienfeld, Germany). The cells were incubated at the same condition until they attached to the glass slips. The cultured epithelial cells seeded on glass coverslips could be applied to intracellular pH measurement and immunohistochemistry. In the extracellular pH measurement, the cultured epithelial cells (5∼6×10^5^ cells/ml) incubated in EMEM culture medium were seeded in the transwells (Corning-Costar Corp., Corning, NY) at 32°C in a humidified atmosphere of 5% CO_2_/95% O_2_. After 2 days of culture, the culture medium in both apical and basolateral side of the cultured epithelium was replaced with fresh EMEM culture medium. The cells were used for extracellular pH measurement after 4 days of culture.

### Immunohistochemistry

Cultured cells were seeded on glass coverslips for 3–4 days. Then the caput epididymal epithelium was washed by PBS three times, and incubated in 4% paraformaldehyde at 4°C for 20 minutes. Rinsed by PBS for three times, the cells were put into 0.1% Triton X-100 PBS solution (PBT) for 5 minutes. The cells were then put into PBT containing 1% Bovine Serum Albumin (BSA) for 30–40 minutes to avoid nonspecific immunofluorescent binding. The cells were incubated in PBT solution containing anti pan keratin antibody (1∶100) (Boster Bioengineering Co., Wuhan, China) for 2 hours at room temperature. After the incubation of the primary antibody, the cells were rinsed with PBS three times to remove the unbinding antibody and incubated in PBS containing secondary antibody-FITC (Boster Bioengineering Co., Wuhan, China) for 1 hour at room temperature. After being washed by PBS for three times, the cells were covered by Vectashield (Vector Laboratories, Burlingame, CA) and 0.3 M Tris buffer (1∶1) against fluorescence quenching. The observation was performed by the laser confocal imaging microscopic system (TCS SP2; Leica microsystem, Germany), in which the excitation wavelength was 488 nm, and the emission wavelength was 515–540 nm. For negative control, the primary antibody was omitted in the same protocol.

### Intracellular pH measurement

Three days after culture, the caput epididymis epithelium cultured on the glass coverslips were washed with HEPES (HCO_3_-free) KH solution three times, and then incubated in HEPES-KH solution containing the dual emission pH sensitive probe SNARF-4f (10 µM, pKa∼6.4, Molecular Probes, Eugene, OR) for 1 hour in the dark at room temperature. After the incubation, the culture cells were washed three times by HEPES-KH solution and then transferred into a homemade chamber coupling with perfusion system (ALA-VM8, ALA Scientific Instruments, Westbury, NY). The chamber was laid in Laser Scanning Confocal Microscopy for pHi measurement. The exciting wavelength of SNARF-4f was 488 nm, and the emission wavelengths were 580 nm and 640 nm. The fluorescence intensity rate of two emission wavelengths was recorded by Leica SP2 matched software, which reflected the changes in pHi. The fluorescence intensity ratios were calibrated in situ by equilibrating the cells in high potassium solutions containing nigericin (Invitrogen Molecular Probes, Eugene, OR) [43]. In the calibration, 131.8 mM NaCl in KH solution was replaced by equal mole KCl and adjusted them to the pH of 6.0, 6.5, 7.0 and 7.5. First incubated the cells with 10 µM nigericin for 10 minutes and then exposed the cells with different pH of high K^+^ KH solution with nigericin for 10 minutes respectively. The ratios of fluorescence intensity in different emission wavelengths were recorded and the linear calibration was made by linear regression

### Extracellular pH measurement

The caput epididymal epithelium cultured on the 0.4 µM pore size and 12 mm diameter transwell (Corning coastar corporation, Cambridge, MA) would form monolayer after 4 day culture. By then, the EMEM culture medium in both sides of the cultured epithelium was changed with Cl^−^-free KH solution. 1 mM DIDS were applied into the Cl^−^-free KH solution of apical side or basolateral side, and the cells were then incubated at 32°C in the humidified atmosphere of 5% CO_2_/95% O_2_ for 1 hour. After that, the thin-bulbed pH electrode (Orion 8163BNWP, Thermo Scientific, Basel, Switzerland) linked to the pH meter (Orion 3 star, Thermo Scientific, Basel, Switzerland) was put into the solution of apical side of the caput epididymal epithelium and the pH values were recorded. Calibration of the thin-bulbed pH electrode was performed following the instructions of the company before each experiment.

### Statistical analysis

All data were presented as mean ± S.E.M.. n represented the repeated number of times refer to animal replicates in each analysis. The unpaired student's t test was applied for comparison between groups. P values<0.05 were accepted as significant.
